# Spinal infection caused by *Aspergillus terreus* in immunocompetent individuals: a case report and literature review

**DOI:** 10.3389/fmed.2025.1701261

**Published:** 2025-12-08

**Authors:** Lingling Liu, Meijuan Pei, Yijie Ma, Xu Guo, Shuai Guo, Haolin Sun, Junqi Zheng, Xueyuan Su, Lihua Meng, Xiaodong Zhang

**Affiliations:** 1 Department of Clinical Laboratory, Peking University First Hospital Taiyuan Hospital/Taiyuan Central Hospital, Taiyuan, China; 2 Peking University First Hospital Taiyuan Hospital/Taiyuan Central Hospital, Taiyuan, China; 3 Fenyang College of Shanxi Medical University, Fenyang, China; 4 Department of Orthopedics, Peking University First Hospital, Beijing, China

**Keywords:** fungi, spinal infection, *Aspergillus terreus*, mycosis, voriconazole

## Abstract

Spinal infections, particularly those caused by *Aspergillus terreus*, represent complex and rare clinical challenges. They are even less common in patients with a normal immune system. This case report details the complex diagnosis and treatment process of a 52-year-old female with *A. terreus-*induced spinal infection. Diagnosis was initially delayed due to non-specific clinical manifestations and ambiguous imaging findings. After treatment with voriconazole (a targeted antifungal agent), the patient’s clinical symptoms and imaging indicators improved significantly—this fully demonstrates the effectiveness of voriconazole in treating such specific fungal spinal infections. This case highlights that fungal infection should be considered in immunocompetent patients with suspected spinal infection, and emphasizes the importance of timely diagnosis and individualized targeted antifungal therapy.

## 1 Introduction

Common fungi encompass eight main categories: *Aspergillus, Mucorales, Fusarium, Scedosporium, Paecilomyces, Penicillium, Dematiaceous fungi, and dimorphic fungi* (e.g., *Talaromyces marneffei* and *Histoplasma capsulatum*), among which *Aspergillus* and *Mucorales* are the most prevalent etiologic agents of invasive fungal infections. The increased prevalence of fungal infections is attributed to the widespread use of immunosuppressants and advances in fungal detection techniques (1).

Fungal infections of the spine represent between 0.5 and 1.6% of all spinal infections (1), and account for approximately 50% of all spinal fungal infections (SFI) (1). The symptoms, signs and laboratory findings of SFI are similar to those of other types of vertebral infections, with no specific imaging characteristics. SFI are exceedingly uncommon in clinical settings and are readily overlooked in laboratory tests, resulting in misdiagnoses or missed diagnoses. This paper provides a comprehensive account of the clinical manifestations, laboratory tests and imaging findings associated with a case of fungal spondylitis in our hospital. It also presents an analysis of 41 clinical cases of fungal spondylitis infection, with the objective of advancing the clinical understanding of the disease and enhancing the detection in hospital laboratories.

## 2 Case presentation

A 52-year-old female was admitted to our hospital on April 15, 2023. Seven months before admission, she had an accidental fall, followed by the onset of lower back pain that gradually exacerbated. Clinical examination suggested a suspected L5 vertebral avulsion fracture, and she received conservative treatment for 3 months. During this period, she had a single episode of fever with a maximum temperature of 40 °C. Despite conservative treatment, severe lower back pain persisted. Subsequent lumbar computed tomography (CT) and magnetic resonance imaging (MRI) scans revealed progressive collapse of the L4-5 intervertebral space and sub-endplate bony destruction, suggestive of a lumbar infection. The patient received lumbar brace fixation and empirical therapy with vancomycin and imipenem for 2 weeks, followed by oral linezolid for 10 weeks, but no improvement in clinical symptoms was observed.

**Figure 1:**
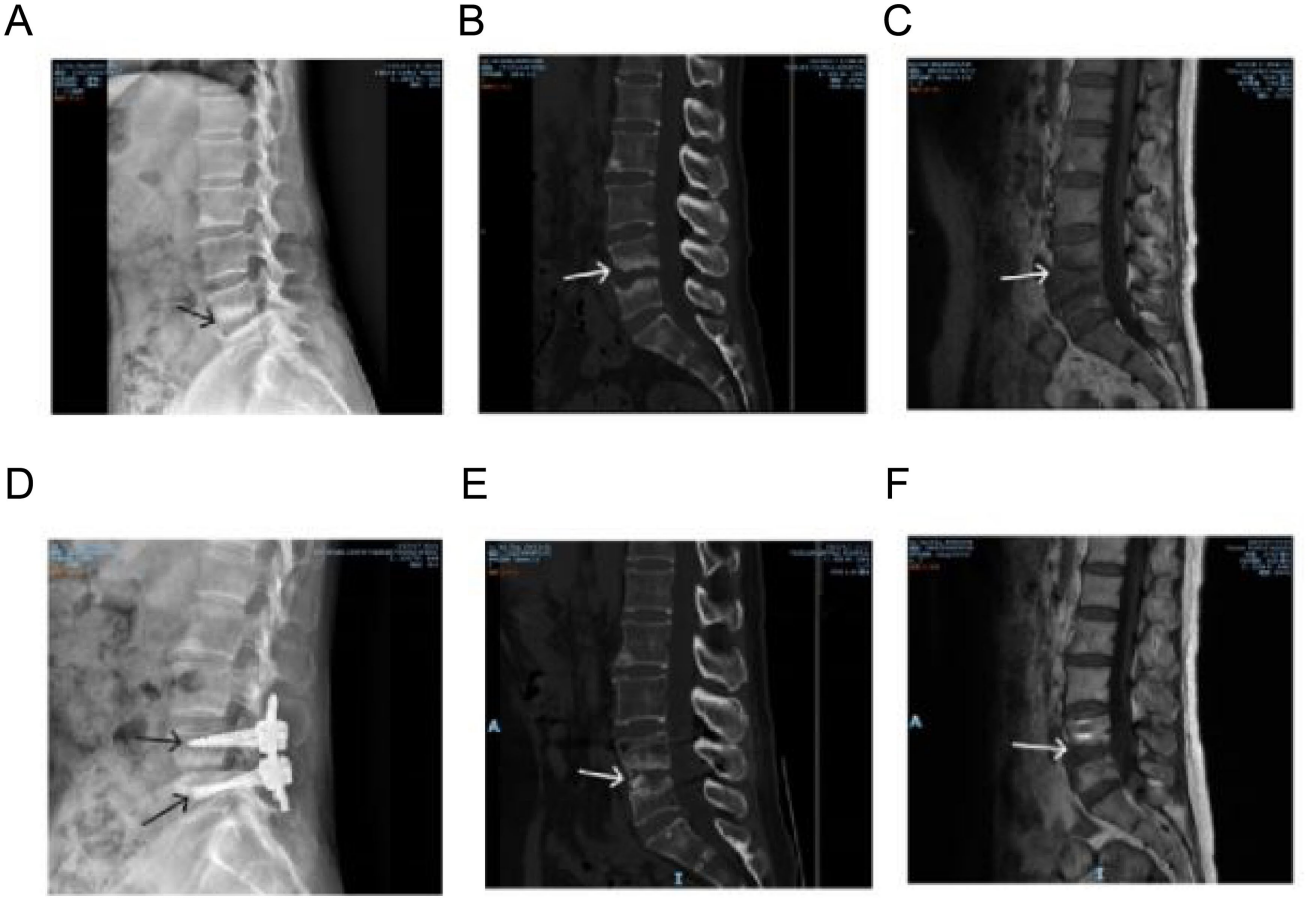
Imaging studies. **(A)** Preoperative X-ray image showing irregularities at the relative edges of L4-5 vertebrae, increased bone density, and mild posterior displacement of the L4 vertebra. **(B)** CT imaging reveals bone loss at the relative edges of the L4-5 vertebrae and increased density. **(C)** MRI image suggesting the presence of local bone loss. **(D–F)** Postoperative imaging at 18 weeks (X-ray, CT, MRI) shows no abnormal signals at the lesion site, indicating no recurrence of infection.

The patient had no history of chronic diseases (e.g., hypertension, diabetes), allergies (drugs/food), tuberculosis, contact with cattle or sheep, or previous surgery. Inspection: Physiological curvature of the spine was present; lumbar range of motion was limited. Palpation: Paravertebral tenderness and percussion tenderness at the L4-5 level were positive; superficial cutaneous sensation of both lower extremities was normal. Motion Examination: Muscle strength of the bilateral iliopsoas, quadriceps femoris, tibialis anterior, extensor hallucis longus, and plantar flexors was Grade V. Muscle tone of both lower extremities was normal; bilateral patellar reflexes and Achilles reflexes were normal. Measurement: Both lower extremities were of equal length. Special Tests: Bilateral straight leg raising test and its augmented test were negative; bilateral “4” sign test was negative; bilateral Babinski signs were negative. Preoperative X-ray showed irregularities at the opposing edges of the L4-5 vertebrae, increased bony density, and mild posterior displacement of the L4 vertebra (Figure 1A). A lumbar CT scan revealed bony defects and increased density at the adjacent margins of the L4 and L5 vertebrae (Figure 1B). An MRI scan revealed hyperintense T1 and T2 signals at the anteroinferior edge of the L2 vertebra, narrowing of the L4-5 intervertebral space, strip-like hypointense T1 and hyperintense T2 signals at the adjacent margins, and possible localized bony defects (Figure 1C). At the 18-week postoperative follow-up, X-ray, CT, and MRI scans showed no abnormal signals in the lesion area (Figures 1D–F), with no signs of infection recurrence.

Peripheral blood tests showed a white blood cell count of 4.34 × 10^9^/L, erythrocyte sedimentation rate (ESR) of 26 mm/h, C-reactive protein (CRP) concentration of 2.1 mg/L, procalcitonin 0.034 ng/mL and interleukin-6 < 2 pg./mL. Tests for Brucella antibodies and agglutination were negative, as were the tuberculosis infection T-cell (T-spot) test, the (1,3)-*β*-D-glucan test (G test) and the galactomannan test (GM test).

**Figure 2:**
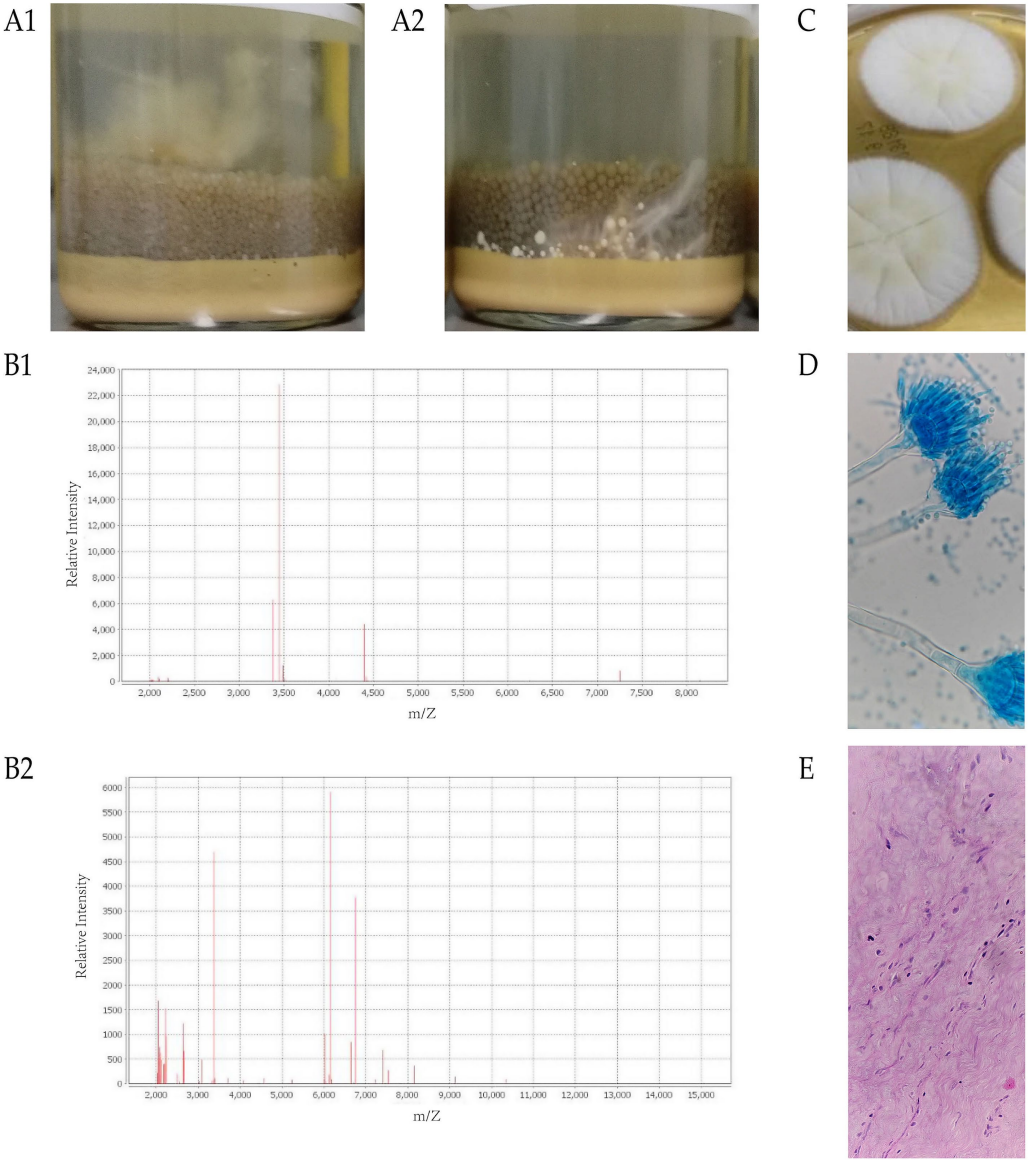
Growth morphology in positive culture bottles. **(A1)** The growth of the culture liquid for Sample 1 after 7.85 days of incubation. **(A2)** The growth of the culture liquid for Sample 2 after 8.9 days of incubation. **(B1)** The mass spectrometry identification results for Sample 1. **(B2)** The mass spectrometry identification results for Sample 2. **(C)** Colony morphology on SDA after 5 days of incubation. **(D)** Photomicrograph showing the morphology of the mold stained (LPCB, 400×). **(E)** Photomicrograph showing infiltration of inflammatory cells. (HE, 200×). HE, Hematoxylin–Eosin staining.

Three tissue grinders (one as backup) and four Mérieux PLUS enrichment culture bottles (two for aerobic and two for anaerobic culture) were prepared in advance and strictly sterilized per supply room surgical instrument protocols. Each tissue sample was ground with a dedicated grinder on a sterile operating room workbench. The aerobic blood culture bottle for Tissue 1 demonstrated positive growth after 7.85 days, accompanied by the observation of visible cotton-like material floating in the bottle (Figure 2A1). The aerobic blood culture bottle for Tissue 2 showed positive growth after 8.9 days, accompanied by the observation of visible cotton-like material adhering to the bottom of the bottle (Figure 2A2). Both anaerobic culture bottles remained negative after 15-days of incubation. Mass spectrometry detection was performed on the flocculent precipitate in the enrichment culture bottle, and the identification result showed that the microorganism was *A. terreus* (Figures 2B1,B2). Meanwhile, a sterile inoculating loop was used to pick a small amount of flocculent precipitate, which was then inoculated onto Columbia Blood Agar (CBA), MacConkey Agar (MAC), and Sabouraud Dextrose Agar (SDA, Zhengzhou Antu) media for subculture. After 2 days of subculturing, fungal morphology was observed on the SDA plate. The colonies were noted to be white and fluffy, exhibiting radial folds and no exudate (Figure 2C). Colonies were picked from the SDA plate using transparent tape, stained with lactophenol cotton blue (LPCB), and the morphology of the fungi observed under a microscope is shown in Figure 2D. Under the microscope, the light microscopy showed typical *Aspergillus* morphology: double-layered conidiophore, that form only in the upper half of the vesicle, resembling a “Mohawk hairstyle.” Antifungal susceptibility testing was performed on *A. terreus* in accordance with the operating instructions of the YeastOne kit (Trek Diagnostic Systems Ltd). The results of the antifungal susceptibility testing are presented in Table 1.

**Table 1:** Antifungal susceptibility test results.

Antifungal agent	MIC (mg/L)	Breakpoint
Anidulafungin	4	None
Micafungin	8	None
Caspofungin	8	ECV 0.12
5-Fluorocytosine	0.12	None
Posaconazole	0.06	ECV 1
Voriconazole	0.12	ECV 2
Itraconazole	0.06	ECV 2
Fluconazole	256	None
Amphotericin B	8	ECV 4

“None” indicates that there is currently no established standard for interpretation.

ECV, epidemiological cutoff value; MIC, minimum inhibitory concentration.

A portion of the tissue was sent to the pathology department for relevant pathological investigations. The pathological findings are illustrated in Figure 2E, indicating degenerative changes in the transparent cartilage and fibrocartilage of the L4-5 intervertebral disc and endplate. These changes include focal proliferation of chondrocytes and mild chronic inflammatory cell infiltration around the small blood vessels in the fibrocartilage.

**Figure 3:**
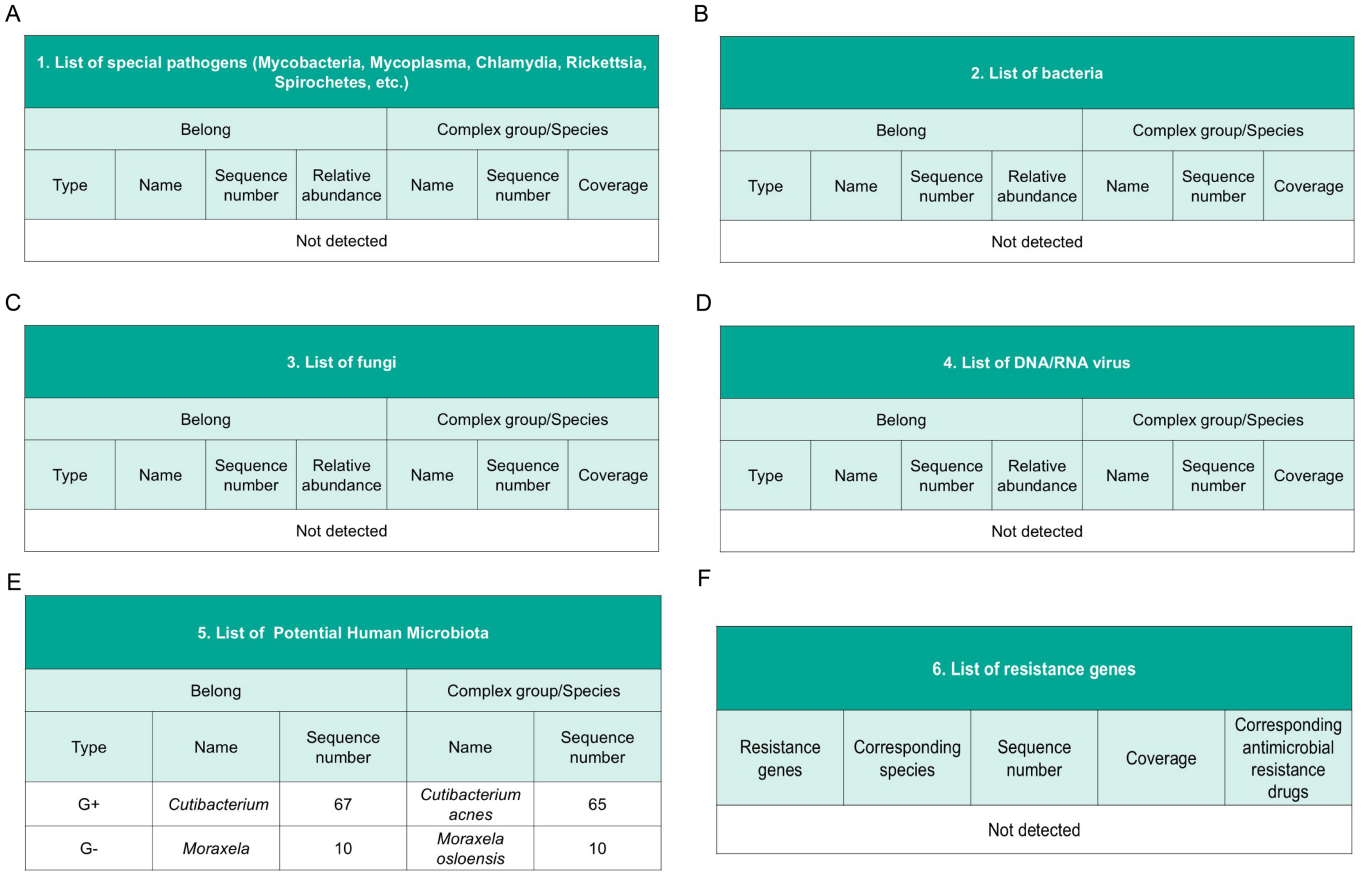
Metagenomic sequencing. **(A)** List of special pathogens. **(B)** List of bacteria. **(C)** List of fungi. **(D)** List of DNA/RNA virus. **(E)** List of potential human microbiota. **(F)** List of resistance genes.

A separate tissue sample was placed in a sterile container for the comprehensive pathogen metagenomic test. This testing was conducted by a third-party laboratory (Kingdom Medical). No evidence of specific pathogens (*mycobacteria, mycoplasma, chlamydia, rickettsia, spirochetes*) was detected; nor were bacteria, fungi, DNA/RNA viruses, parasites, or drug resistance genes identified. However, Human microbiota such as Moraxella, spp., Cutibacterium, spp., Cutibacterium acnes, *Moraxella osloensis* were detected (Figure 3).

## 3 Literature review on fungal spinal infections

A comprehensive review of the literature was conducted, and 41 clinical cases of mold spondylitis from 1998 to 2025 analysed. The summarized information is given in Table 2.

**Table 2:** Retrospective analysis of 40 cases of mold spondylodiskitis.

Number	Sex/age	Predisposing factor (s)	ESR (mm/h)	Location	Causative organism	Diagnostic methods
(1)	F/42	Yes	90	T2-4	*A. fumigatus*	Microbial cultivation; pathologic diagnosis; molecular biological detection
(2)	M/67	Yes	96	T1-8	*A. fumigatus*	Microbial cultivation
(2)	M/68	Yes	34	T12-L2	*A. fumigatus*	Microbial cultivation; pathologic diagnosis
(2)	F/50	Yes	115	L3–4	*A. fumigatus*	Microbial cultivation; pathologic diagnosis
(2)	M/48	No	49	L4-5	*A. fumigatus*	Microbial cultivation; pathologic diagnosis
(2)	M/43	Yes	32	L4-5	*A. niger*	Microbial cultivation
(2)	M/57	No	30	L5-S1	*A. fumigatus*	Pathologic diagnosis
(9)	M/35	Yes	48	L4-5	*A. fumigatus*	Microbial cultivation
(10)	F/62	No	41	T10-11	*A. fumigatus*	Microbial cultivation; pathologic diagnosis
(11)	M/69	Yes	–	T12-L1	*A. fumigatus*	Microbial cultivation
(11)	F/39	Yes	–	L4-5	*A. fumigatus*	Microbial cultivation
(12)	F/39	Yes	60	L5-S1	*A. fumigatus*	Microbial cultivation
(12)	F/33	Yes	80	L4-5	*A. fumigatus*	Microbial cultivation
(13)	F/38	No	43	T4-7	Mold	Pathologic diagnosis
(14)	F/70	Yes	–	S3-4,6	*A. fumigatus*	Microbial cultivation; molecular biological detection
(15)	M/50	Yes	–	T2-8	*A. fumigatus*	Pathologic diagnosis
(16)	F/31	Yes	–	T12-L1	*A. fumigatus*	Microbial cultivation
(17)	M/17	Yes	113	T2-3	*A. fumigatus*	Microbial cultivation
(18)	M/74	No	–	T11-12	*A.s terreus*	Serological testing
(19)	F/33	Yes	110	L2-3	*A. fumigatus*	Microbial cultivation
(20)	M/64	Yes	120	L4-5	*Mucorales*	Microbial cultivation; pathologic diagnosis
(21)	M/59	Yes	96	L3-4	*Aflatoxin*	Molecular biological detection
(22)	M/17	Yes	–	L3-4	*Aflatoxin*	Microbial cultivation; pathologic diagnosis
(23)	M/60	Yes	>100	T11-12	*Aspergillus*	Pathologic diagnosis
(24)	F/34	Yes	–	C6-7, C7-T1	*Aspergillus*	Microbial cultivation; pathologic diagnosis
(25)	F/40	Yes	48	T1-3	*A. nidulans*	Microbial cultivation; molecular biological detection
(26)	F/58	Yes	–	T11-12	*Aspergillus*	Pathologic diagnosis
(27)	M/76	Yes	55	T6-7	*A. fumigatus*	Microbial cultivation
(27)	M/47	Yes	70	T10-11	*A. fumigatus*	Microbial cultivation; pathologic diagnosis
(28)	M/68	Yes	–	T12/L1-2	*A. fumigatus*	Microbial cultivation
(29)	F/35	No	47	T9-10	*Aspergillus*	Microbial cultivation
(30)	M/48	Yes	–	T4-5	*Mucorales*	Pathologic diagnosis
(31)	M/13	Yes	↑	T11-12	*A. fumigatus*	Microbial cultivation; pathologic diagnosis
(32)	F/57	Yes	104	L4-5	*Rhizopus*	Microbial cultivation
(33)	M/10	Yes	75	T4-5	*A. nidulans*	Microbial cultivation; pathologic diagnosis
(8)	M/25	No	–	L4-5	*Aspergillus*	Microbial cultivation
(8)	M/19	No	–	T10-11	*Aspergillus*	Microbial cultivation; pathologic diagnosis
(34)	F/45	Yes	N	L5-S1	*A. fumigatus*	Microbial cultivation; pathologic diagnosis
(35)	M/70	Yes	–	L2, L4, L5-S2	*Aspergillus*	Microbial cultivation; pathologic diagnosis
(36)	F/39	Yes	–	L4-5	*A. terreus*	Microbial cultivation
The present case	F/52	No	26	L4-5	*A. terreus*	Microbial cultivation

“–,” no date; “↑,” raised.

C, cervical; ESR, erythrocyte sedimentation rate; L, lumbar; F, female; M, male; S, sacral; T, thoracic.

## 4 Discussion

Fungal spondylitis is a clinical condition that is highly prone to being overlooked or misdiagnosed (2). In recent years, the detection rate of pathogens in specimens from sterile sites by clinical microbiology laboratories has increased significantly, which is mainly attributed to three factors: first, the optimization of traditional culture and identification techniques; second, the widespread application of the G test [(1,3)-*β*-D-glucan test] and the GM test (galactomannan test); and third, the growing popularity of metagenomic next-generation sequencing (mNGS) technology. Against this backdrop, the detection rate of fungal spondylitis has also increased remarkably.

Pre-analytical management of specimens covers three key processes: collection, transportation, and preliminary processing. Studies have shown that errors occurring in the pre-analytical phase account for 46%–68.2% of the total errors throughout the testing process (3). This indicates that the correct collection of specimens is crucial for the accuracy of microbiological test results, a key point that clinicians, nurses, and laboratory technicians must fully grasp. Particularly during the preparation of spinal tissue specimens, efficient communication between clinical microbiology technicians and surgeons is even more critical.

The laboratory testing methods and procedures for fungal infections are relatively standardized, with core approaches including direct microscopic examination, culture, serological testing (e.g., G test, GM test, and *Aspergillus* IgG antibody detection), and molecular biology testing (e.g., PCR and mNGS). In the serological diagnosis of fungal infections, the G test and GM test hold certain clinical significance. However, the infusion of certain specific blood products or immunoglobulins may lead to false positive results in the G test. The GM test specifically detects *Aspergillus* antigens, yet a variety of factors can cause it to yield false positive or false negative results. With the advancement of molecular biology techniques, the metagenomic next-generation sequencing (mNGS) technology—used for the identification of fungal infections and detection of drug resistance genes—has emerged as a significant advancement in this field, by virtue of its advantages of rapid detection speed and high specificity. Nevertheless, mNGS also faces certain challenges: on the one hand, its detection cost is relatively high; on the other hand, the thick cell wall of fungi makes DNA extraction difficult, which may result in false negative results. For further identification of the fungal species, macroscopic and microscopic morphological observation, mass spectrometry analysis, and molecular biology techniques may be combined; while *in vitro* antifungal susceptibility testing can offer valuable guidance for the clinical selection of optimal therapeutic regimens (4).

.This study retrospectively analyzed 40 cases of fungal myelitis. The results showed that the male-to-female ratio of the patients was 1.24:1, with an average age of 46.55 years. The etiological test results were as follows: 21 cases of *A. fumigatus*, one case of *A. niger*, two cases of *A. terreus*, two cases of *Mucor*, two cases of *A. flavus*, two cases of *A. nidulans*, one case of *Rhizopus*, and the remaining eight cases were other *Aspergillus* species. Additionally, scholars such as Gamaletsou et al. (5) analyzed 180 cases of *Aspergillus* spondylodiscitis and found that the positive rate of direct tissue culture for *Aspergillus* was 87%, the positive rate of histopathological examination was 64%, and the positive rate of combined detection with both methods was 52% (5). These data fully illustrates the necessity of submitting tissue samples for multiple tests simultaneously.

From the perspective of pathogenesis, the occurrence of fungal diseases is mostly related to the inhalation of fungal conidia in the air (6). For people with normal immune function, the immune system can resist and eliminate fungi through innate and adaptive immune mechanisms, among which alveolar macrophages and neutrophils are the main immune cells involved (6). These two cell types can phagocytose and destroy developing conidia through various mechanisms; within 24 h, they can eliminate fungal cells via the action of acidified phagolysosomes and the production of NADPH oxidase-dependent reactive oxygen species (6). However, in immunocompromised individuals, inhaled conidia may evade immune surveillance and proliferate in the lungs (6); in the later stages, fungal hyphae may invade pulmonary arterioles and lung parenchyma, causing lung necrosis, and even hematogenous dissemination (6). In the present case, we hypothesize that the infection originated as an opportunistic infection following the patient’s fall, given that both environmental surface cultures and air cultures of the operating room on the day in question yielded negative results. Furthermore, two separate tissue samples obtained from the same anatomical site were cultured independently, and both consistently identified *A. terreus*.

Scholars such as Jin and Yin (7) proposed that acupuncture may induce fungal infections. It should be noted that the interval from infection to symptom onset of fungal spondylitis is relatively long, and back pain caused by infection is not common. In clinical practice, when patients seek medical treatment for back pain, doctors often use acupuncture for treatment based on experience, but at this time, the patients may have been infected with the relevant pathogens before acupuncture.

In clinical practice, fungal spondylitis is also often misdiagnosed as tuberculous spondylitis (8). In the present case, the patient was treated empirically for tuberculosis and took oral linezolid for 10 weeks, yet there was no improvement in their clinical symptoms. Testing for tuberculosis using T-cell testing, X-pert testing of tissue specimens and mNGS all yielded negative results, confirming that the patient did not have a *Mycobacterium tuberculosis* infection.

The treatment for fungal spondylitis comprises surgical removal of the infected tissue, followed by antifungal therapy. *A. terreus* is naturally resistant to fluconazole and polyene antifungals (such as amphotericin B). In accordance with THE SANFORD GUIDE TO ANTIMICROBIAL THERAPY 2020 (50th Edition), the use of voriconazole or isavuconazole is recommended for at least 6 months. In this case, the patient was given a 6-month course of voriconazole treatment. Subsequent tests, including complete blood counts, liver and kidney function tests, ESR and CRP all returned normal results. X-ray imaging revealed optimal positioning and morphology of the internal fixation, while computed tomography scans showed that successful bone fusion had occurred in the intervertebral space, accompanied by well-developed bone formation and reconstruction. MRI revealed no abnormal signals at the lesion site, indicating that the infection had not recurred and that a clinical cure had been achieved. A review of the existing literature showed that the mortality rate for fungal spondylitis is 53%, with only 26% of patients achieving complete recovery, likely reflecting a population with compromised immunity. In contrast, our case involved a patient without severe underlying diseases and with normal immune functions, who achieved clinical cure following standardized surgical intervention and antifungal treatment.

During this study’s antifungal susceptibility testing for *A. terreus*, there were no available standards for anidulafungin, micafungin and 5-flucytosine, which precluded the possibility of providing clinical recommendations for these three drugs. The literature review included limited case data on the G and GM tests, which made it challenging to objectively assess the diagnostic value of these tests for fungal spondylitis.

## 5 Conclusion

The culturing and detection of SFI provides certain challenges that can easily result in missed diagnoses. This study employed the use of at least two tissue samples, which were ground and cultured under strict sterile conditions in the operating room, followed by direct mass spectrometry identification of microorganisms after positive results from the enrichment bottles, providing indispensable evidence for clinical diagnosis and treatment. In clinical practice, when there is suspicion of a spinal infection based on imaging results, an early biopsy or surgical intervention should be performed. A combined strategy involving microbial smear, enrichment culture, pathological biopsy, and mNGS—applied to surgical specimens—holds great potential for significantly improving the pathogen detection rate, thereby providing robust support for the timely and accurate diagnosis and subsequent treatment of patients.

## Data Availability

The raw data supporting the conclusions of this article will be made available by the authors, without undue reservation.
